# Application of High-Throughput Screening Raman Spectroscopy (HTS-RS) for Label-Free Identification and Molecular Characterization of Pollen

**DOI:** 10.3390/s19204428

**Published:** 2019-10-12

**Authors:** Abdullah S. Mondol, Milind D. Patel, Jan Rüger, Clara Stiebing, Andreas Kleiber, Thomas Henkel, Jürgen Popp, Iwan W. Schie

**Affiliations:** 1 Leibniz Institute of Photonic Technology, Albert Einstein Str. 9, 07745 Jena, Germany; abdullah.mondol@leibniz-ipht.de (A.S.M.); patelmilind2993@gmail.com (M.D.P.); jan.rueger@leibniz-ipht.de (J.R.); clara.stiebing@leibniz-ipht.de (C.S.); andreas.kleiber@leibniz-ipht.de (A.K.); thomas.henkel@leibniz-ipht.de (T.H.); juergen.popp@ipht-jena.de (J.P.); 2 Institute of Physical Chemistry and Abbe Center of Photonics, Friedrich-Schiller University Jena, Helmholtzweg 4, 07743 Jena, Germany; 3 Department of Medical Engineering and Biotechnology, University of Applied Sciences Jena, Carl-Zeiss Promenade 2, 07745 Jena, Germany

**Keywords:** Raman spectroscopy, high throughput screening, pollen detection, PCA-SVM, HCA

## Abstract

Pollen studies play a critical role in various fields of science. In the last couple of decades, replacement of manual identification of pollen by image-based methods using pollen morphological features was a great leap forward, but challenges for pollen with similar morphology remain, and additional approaches are required. Spectroscopy approaches for identification of pollen, such as Raman spectroscopy has potential benefits over traditional methods, due to the investigation of the intrinsic molecular composition of a sample. However, current Raman-based characterization of pollen is complex and time-consuming, resulting in low throughput and limiting the statistical significance of the acquired data. Previously demonstrated high-throughput screening Raman spectroscopy (HTS-RS) eliminates the complexity as well as human interaction by incorporation full automation of the data acquisition process. Here, we present a customization of HTS-RS for pollen identification, enabling sampling of a large number of pollen in comparison to other state-of-the-art Raman pollen investigations. We show that using Raman spectra we are able to provide a preliminary estimation of pollen types based on growth habits using hierarchical cluster analysis (HCA) as well as good taxonomy of 37 different Pollen using principal component analysis-support vector machine (PCA-SVM) with good accuracy even for the pollen specimens sharing similar morphological features. Our results suggest that HTS-RS platform meets the demands for automated pollen detection making it an alternative method for research concerning pollen.

## 1. Introduction

Pollen are the male gametophyte of higher plants enabling fertilization of plants via pollination. The study of pollen can reveal the origin of flora and provide information about insect migration and climate change [1,2]. Moreover, detection and analysis of pollen benefit the study of allergy-related respiratory health issues [3,4,5,6]. An important aspect of pollen research is to identify pollen specimens fast, reliably, and automatically [7,8]. The requirements also demand consistent identification of a significant amount of pollen samples, while minimizing the intra and inter specimen bias. The bias is related to the deviations or variations that occur while identifying a particular pollen sample. For example, the intra and inter specimen bias occurs when pollen samples are collected from the same site and different sites, respectively. As most pollen exhibit distinctive visual features, professional palynologists frequently classify them by characterizing brightfield microscopy images. Because this process is often performed manually, it results in a low number of samples with questionable consistency. There have been significant developments to fully automate this process, by performing image-based identification [7,9,10,11,12,13]. This image-based pollen identification can rapidly classify a large number of pollen specimens, while immensely reducing human dependency. In many situations, for example for grain samples, where samples have similar morphological features, a purely visual differentiation approach can become quite challenging. Spectroscopic investigation of pollen samples to address this issue was already explored, e.g., Fourier-transform infrared (FTIR) spectroscopy [1,2,3,4,14,15,16,17], laser-induced breakdown microscopy [18,19], fluorescence spectroscopy [14,20], attenuated total reflectance [2,17], high performance thin layer chromatography [21], and Raman spectroscopy [22,23,24,25,26,27]. 

Fluorescence measurements perform better for bulk samples with multiple excitation wavelengths [14] in contrast to the investigation of each pollen grain with a single excitation wavelength [20], rendering only limited molecular information and require additional sample preparation steps. Proof of concept for on-line automatic atmospheric pollen quantification has also been proposed recently using fluorescence and light scattering techniques [28,29]. Vibrational spectroscopy (FTIR and Raman spectroscopy) provides significantly higher molecular information label-free. FTIR is fast and free from fluorescent contributions, but cannot be used in aqueous solutions [4]. As a complementary method, Raman spectroscopy allows measurements in both aqueous solution and air with high spectral resolution.

Raman spectroscopy has readily been used on eukaryotic and prokaryotic cells, for example, to study drug cell interaction [30], cell cycle analysis [31], and cell toxicity [32]. Pollen samples have also been extensively investigated using different Raman modalities, i.e., spontaneous Raman scattering [5,6,21,23,26], resonance Raman scattering [21], surface-enhanced Raman scattering [24,27], and FT-Raman spectroscopy [17,22]. The advantage of Raman spectroscopy is that there is no particular need for sample preparation and the assessed information reflects the intrinsic molecular signature of the sample [21,23,26]. Although Raman measurements on pollen are commonly conducted in a water environment, they can be also investigated in air [6,19,20,25]. The optimal experimental conditions for pollen Raman measurements were evaluated and discussed in [20]. A majority of publications for the characterization of pollen using Raman spectroscopy are carried out using Raman band analysis, which has been shown to suffice in many simple cases [5,15,19,22,23,25,26,27]. Ivleva et al. correlated various macro-molecules (protein, lipid, carbohydrates, nucleic acid, and carotenoids) to Raman spectral features [5] whereas, Schulte et al. investigated the Raman spectral signature associated with germination [26]. Moreover, the implementation of multivariate statistics ensures robust and correct taxonomical identification of pollen using various methods, namely, hierarchical cluster analysis (HCA) [5,17,23,24], principal component analysis (PCA) [2,5,17,24], and others [6,24]. Schulte et al. identified 15 pollen species using HCA with less than 4% misclassification [23], whereas Seifert et al. used artificial neural networks (ANN) and demonstrated that it performs better than HCA and PCA [24]. 

In spite of all the advantages and impressive performances, conventional Raman systems are not the premiere choice for pollen measurements, as it usually requires intensive human labor, resulting in long experimental times and limited sample sizes. Recently, we have reported on the development of a high throughput screening Raman spectroscopy (HTS-RS) platform to measure thousands of eukaryotic cells in a short period of time [33]. To overcome the current limitation of spectroscopic characterization of pollen, we have extended our platform for the investigation and characterization of pollen samples, and we were able to significantly improve the current state-of-the-art for molecular pollen profiling. 

In this work, we used the improved HTS-RS platform to measure 37 different pollen species. We were able to measure a significant number of pollen samples from each specimen completely automatically with minimum human interaction, ensuring high-throughput and robust statistical analysis. By applying multivariate statistical methods, we were able to screen pollen types according to their growth habit with comparable performances to other studies. Using a PCA-SVM model, it was possible to identify pollen genus and to establish their taxonomy. Our results indicate that the developed method meets the demands of automated palynology. More precisely, the presented approach can measure a large number of samples in comparison to conventional Raman measurements and provides taxonomical resolution based on specific chemical signatures, making the HTS-RS a competitive approach to the current state-of-the-art techniques.

## 2. Materials and Methods

### 2.1. Sample Collection

In total, 37 different pollen species (Table 1), which were donated by the Department of Indoor Climatology, University Hospital Jena, Germany, to the Leibniz-IPHT, investigated in the present study. 

### 2.2. HTS-RS Platform 

**Figure 1:**
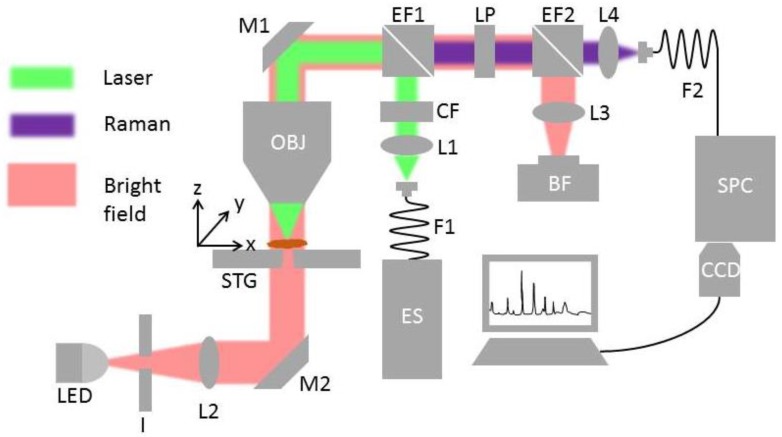
Illustration of the experimental setup for Raman spectroscopic measurements. The three paths: Raman excitation, Raman collection, and bright field illumination are represented in green, purple and pink, respectively. The components are ES: Excitation source, F: Optical fiber, L: Lens, CF: Clean-up filter, EF: Edge filter, M: Mirror, OBJ: Objective, STG: Motorized stages, I: Iris aperture, LED: Light emitting diode, LP: Long pass filter, BF: Bright field CCD camera, SPC: Spectrograph, and CCD: deep depletion CCD.

The in-house build Raman system has four major functional blocks, which are depicted in Figure 1: Excitation path (green line), collection path (purple line), bright field illumination path (red line), and the motorized stages for automation. The first two functions are responsible for the generation and collection of the Raman signal, whereas the last two are required for visualization and sample translational. The excitation to the system was performed by a fiber-coupled 785 nm excitation laser (ES) (Xtra, Troptica, Germany) and a multimode fiber (F1) (62 µm, 0.22 NA FC-PC, Thorlabs, Germany). It was imaged into infinity using a collimation lens (L1) with a 30 mm focal length (Thorlabs, Germany). After passing a clean-up filter (CF) (Semrock, Germany), to remove signal contributions from the fiber, the beam was directed by an edge filter (EF1) and a 45° mirror (M1) to a 60× NA 1.0 water immersion objective lens (OBJ) (Nikon, Japan). The objective lens focused the excitation beam onto a 10 µm diameter spot in the sample plane. The generated Raman signal and the Rayleigh scattering were collected again by the OBJ, and after passing EF1, separated from the laser excitation light. Due to the high contribution of Rayleigh scattering, an additional long-pass filter (LP) (Semrock, Germany) was used. The Raman signal was focused by a 75 mm focal length lens (L4) onto a 105 µm NA 0.12 multimode fiber (F2), which guided the Raman signal to a spectrograph (SPC) (IsoPlane, Princestone, USA) with f/#4.0 and equipped with 400 lines/mm grating. F2 not only delivered the light to the spectrometer, but also acted as an entrance aperture for SPC. The entrance aperture was imaged onto a back-illuminated deep depletion CCD camera (CCD) (Pixis 400, Princestone, USA), operating at −60°C (Pixis, Princestone, USA). A homogenous-illumination was implemented using a white light-emitting diode (LED), iris aperture (I), lens (L2), and a 45° mirror (M2). The light transmitted through the sample plane and all the optics until EF2, where it was then focused by a 100 mm focal length lens (L3) onto the bright field camera (BF) (DCC1645C, Thorlabs, Germany). The bright-field image was the core for various automation functions, such as autofocus, pollen detection, and sample plane calibration. The sample plane calibration corrected the tilt of slides on the sample holder ensuring proper measurement. The sample holder consisted of three integrated motorized stages for x-y-z translational movement (STG). The x-y translational stages (Newport, USA) allowed to position each pollen sample below the objective lens, while the z-stage assisted in finding the best focal plane for both the Raman signal and bright field illumination. 

### 2.3. Raman Measurement of the Pollen Sample

The measurements of pollen samples were performed on CaF_2_ slides similar to the previous work [33,34] while the key differences are mentioned in the next paragraph. When samples were placed under the OBJ, a bright-field image also termed as field of view (FoV), was acquired. Afterwards, a cell detection algorithm was applied to the bright field image, which detected the particles and their coordinates of each sample in the FoV. The pollen detection scheme is further elaborated in the next subsection. After the localization, the OBJ was translated onto each of the particles using STG while acquiring spectra. When all the particles in a FoV were investigated, the next FoV was investigated in the same way. This whole process was repeated until all initially defined FoVs were measured. The number of field of views (FoV) was kept constant to 10 × 10 frames for each measurement for this study. Before every measurement, to correct the typical tilt of CaF_2_ slides, an in-house developed software algorithm initially estimated the tilt and adjusted it accordingly for each frame or FoV. For this study, an excitation power of 140 mW (λ = 785 nm) and an integration time of 0.5 s were used. With this process, several hundreds of pollen particles from each genus were sampled rapidly.

In this work several system modifications were implemented in comparison to previously reported work [33,34], such as the development of a new image-based localization algorithm for pollen, the improvement of the autofocus function incorporating more points, as well as applying Gaussian curve fitting, the implementation of a homogenous illumination scheme for the bright field image. The improved autofocus function enables locating the best focal plane more precisely than the previous developments in [33,34] ensuring the Raman spectral quality whereas the homogenous illumination significantly improves the particle detection precision.

### 2.4. Pollen Localization from Bright Field Image 

**Figure 2:**
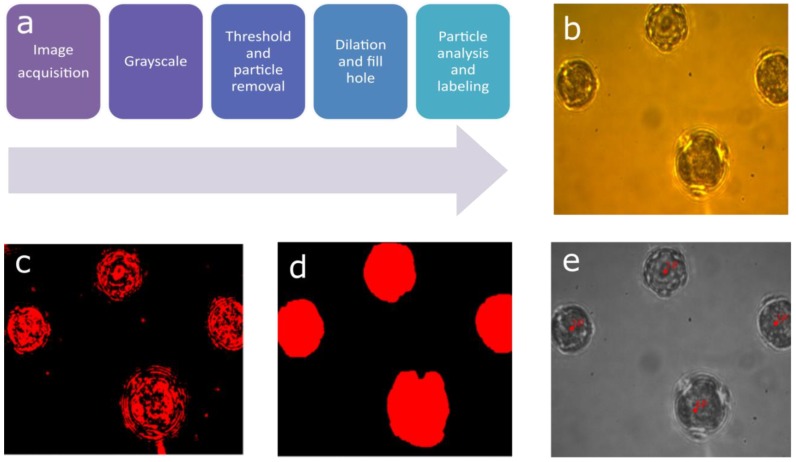
The pollen detection algorithm follows a set of steps (**a**) after acquiring the bright field image (**b**). As a result of grayscale conversion, threshold, and particle removal, a binary image is created (**c**). This binary image converted to fill the holes in particles (**d**). Later a blob analysis is performed to acquire the coordinate of the pollen samples (**e**).

Figure 2 shows the image processing steps to determine the localization of the pollen sample (Figure 2a). At first, the acquired bright field image (Figure 2b) was converted to an 8-bit greyscale image to simplify further processing steps. A calculation of image histogram followed, providing an intensity distribution, which was used to determine a threshold value to separate the pollen from the background in the binary image. As pollen particles have specific sizes, the unwanted particles not corresponding to that were removed by a particle removal step (Figure 2c). The holes and voids of pollen in the binary image were filled by dilation and fill-hole thickening operation. The fill-hole operation applies a structuring element, generally a 3 x 3 window, to the source image and calculates a union or logical-or between them. Conduction of this operation several times removed small voids from the blobs in the transformed image. The dilation operation also worked similarly to the fill-hole operation but used a different structuring element. It extended the binary pixels of the existing bodies allowing more stable blobs, corresponding to each of the pollen. The combination of fill hole and dilation improved blob morphology, resulting in improved separation of pollen from the background (Figure 2d). Last, a blob analysis was performed on the processed image. Blob analysis provided various pieces of statistical information regarding morphologies present in an image. For this study, the number of pollens in a FoV and the corresponding pixel coordinates (Figure 2e) were acquired from blob analysis. The pixel coordinates were converted to motor coordinates facilitating translation to the pollen samples.

### 2.5. Data Analysis of Raman Spectra

Raman spectral data analysis consists of two parts: Spectral pre-processing followed by a multivariate statistical analysis of the preprocessed data. The spectral preprocessing involves multiple steps, i.e., wavelength calibration, intensity calibration, cosmic spike removal, background correction, and area normalization [35]. Wavelength calibration was performed by using 4-acetaminophenol (Sigma-Aldrich, Germany) as a calibration standard [36]. Intensity calibration was performed by recording spectra of a NIST standard (National Institute of Standards and Technology, USA) white light calibration lamp (Kaiser optical system, USA) and correcting for the spectral response function of the system [36]. Extended multiplicative signal correction (EMSC) [37] was used to remove background from endogenous fluorophores and water. A background-corrected pollen spectrum, a water spectrum, first and second-order polynomials were used for EMSC. This method was previously applied for background correction of Raman spectra [2,31,33,38]. The resulting spectra were area normalized. A comparison of mean spectra before spectral preprocessing and afterwards (Suppl. Mat.) shows successful recovery of relevant Raman signals from the measured spectra. 

Several multivariate statistical techniques were carried out to extract meaningful information from the preprocessed Raman data. HCA was applied to the mean spectra of each pollen sample genus to estimate the pollen growth habit. HCA is an unsupervised machine learning method, which computes distances between all objects in the original variable space and groups them into a cluster [39]. As HCA confines discrete clusters [39] between all the acquired spectra in the dataset, it is computationally expensive. Hence, only the mean spectra of the fingerprint region (758–1800 cm^−1^) from 37 different pollen specimens were used. Here, the pairwise distance between the spectra was computed, using the Minkowski distance [40] with an exponent value of five. A hierarchical cluster tree was created using average linkage, resulting in a Cophenetic correlation coefficient [41] of 0.9008. 

Principal component analysis (PCA) combined with a support vector machine (SVM) as a supervised machine learning method was used to determine the pollen taxonomy from the acquired Raman spectra. First, the data set was grouped according to their growth habit type, i.e., grass, herb, shrubs, and tree. Afterwards, PCA was applied to each of the groups or datasets separately considering Raman spectral fingerprint and high wavenumber region. Application of PCA to spectral dataset reduces the dimension of Raman spectra to an orthogonal set of basis vectors known as loadings or principal components (PCs) where the first PCs correspond to the highest variance in the dataset [42,43]. The PCs are orderly structured, which means that the first PC corresponds to the maximum variance, the second PC corresponds to the second most variance and so on. These PCs can be considered as a new axis and for each of the spectra, a corresponding score is assigned. The method enables investigations of minute spectral changes by plotting the scores in a score plot. After the dimension reduction, an SVM model was trained using 100 randomly chosen scores sets, characterizing the pollen. SVM uses optimized hyper-planes, a decision boundary that is estimated by maximizing the margin in between the classes [44]. First, nine PCs, as it corresponds to more than 95% of cumulative spectral variance, were considered for the SVM training. The SVM used one-versus-one coding for a binary model for unique class labels for each of the pollen taxa by employing an error-correcting output codes method. Each of the binary learners of the SVM model used different alpha and bias values using the Gaussian kernel function. Applications of PCA-SVM for Raman spectral data analysis have been already demonstrated [45,46,47]. Before the application of the PCA-SVM model to the rest of the spectral dataset, a ten-fold leave one out cross-validation was performed estimating the model performance by means of average misclassification. For this, the 100 randomly chosen scores from PCA were divided into 10 groups where the nine were used for modeling and the rest was used for prediction. A comparison of the prediction and the original class of training provided an estimate of model performance. Afterwards, the PCA-SVM model was applied to the test data, which was left out from the modeling procedure predicting the pollen genus. The comparison between predicted pollen genera and the corresponding references presented using a confusion matrix. The target and the output class in the confusion represent the actual pollen and the model prediction class, respectively. The number and the percentage value in each of the blocks of the confusion matrix represent the total number points for analysis and their corresponding contribution to the whole data set, respectively. The percentages in green in the last row and column of the matrix compute the whole positive predictive value and sensitivity for each class.

## 3. Results

Four different pollen growth habit types have been investigated from 37 different pollen species. The results of unsupervised and supervised machine learning methods are presented in this section. The unsupervised method HCA estimated the pollen type based on the growth habit whereas the supervised method PCA-SVM predicted pollen taxonomy.

### 3.1. Pollen Type Identification Using Raman Spectra 

**Figure 3:**
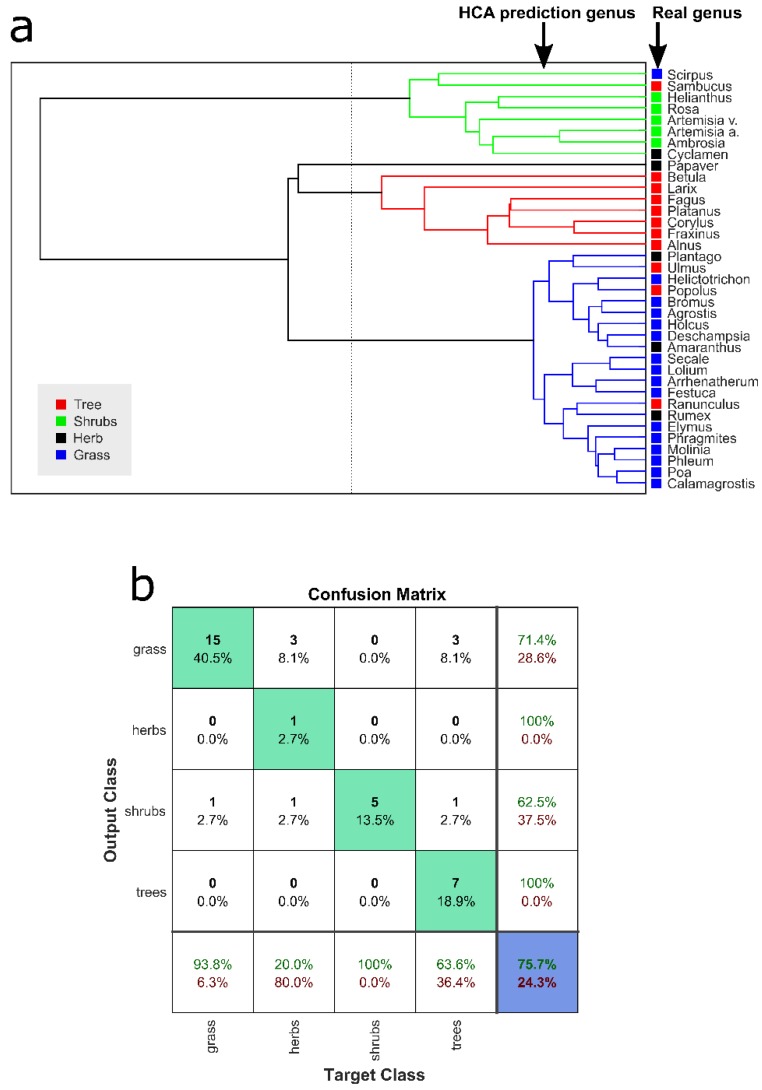
HCA performed on the average pollen samples shows clustering based on its spectral features and is presented as a decision tree indicated by the lines in (**a**). Along the vertical dashed line in the dendrogram, the data is divided into four different groups (output class). Their corresponding actual groups are indicated by the solid squares next to their names. There is a high overlap between real and predicted values. A comparison of is computed in the form of a confusion matrix with an overall accuracy of 75.7% (**b**).

**Figure 4:**
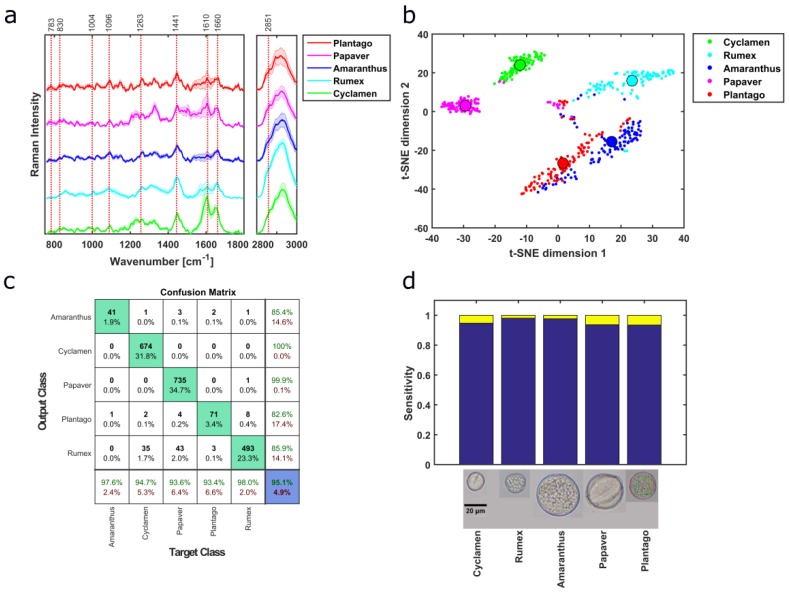
The background-corrected mean spectra of the herb pollen samples (**a**) show distinctive spectral features. After applying PCA to the spectral dataset, the scores are separated into training and test dataset where the t-SNE plot of the first one also demonstrates good separation (**b**). Here, the small dots correspond to each training data point and the big circle shows their median. The output class of the confusion matrix represents the prediction of the PCA-SVM model (**c**). The sensitivities of the PCA-SVM model (the last row green numbers of (**c**)) are compared in a barplot for better visualization (**d**). Here, all the herb pollen samples taxa have a sensitivity of over 95%.

Performing HCA on the mean spectra of all 37 pollen types allowed classifying the pollen according to their growth habit. The cutoff of the HCA cluster, separating the different pollen types, is indicated by the dashed line (Figure 3a). The actual types are shown as solid squares on the right side. A comparison of the actual and predicted pollen types is summarized in the confusion matrix, providing an accuracy of 75.7% (Figure 3b). Although the classification of grasses and shrubs is good, i.e., sensitivity, 93.8% and 100%, respectively, the classification of herbs and trees with a sensitivity of 20% and 63.6%, respectively, is rather poor (Figure 3b), influencing the overall accuracy of the approach. Low sensitivity of herb can be arising due to the confound separation between growth habits of herbs against shrubs and spectral similarities with the grass types (Figures 4a and 6a). For the herb pollen, only one out of four is classified correctly. A spectral similarity of pollen from different subtypes is reported [17,27]. The HCA classification of the grass renders the best performance with only one misclassification of *Scirpus* (Figure 3a), which also has different Raman spectral feature than other samples and can be due belonging to a different family type. The HCA classification of shrubs demonstrates better performance than that of trees and herbs. Guedes et al. [6] categorized the 34 pollen species according to their growth habit into two groups: (1) Trees and shrubs, and (2) grass and herbs. Others have applied HCA for pollen sample classification in the genus and species level [23,24]. Application of HCA to FTIR pollen spectra for taxonomical identification resulted in 84% accuracy [4], which is also comparable to our findings. These results imply that the application of HCA on Raman spectra can provide the preliminary estimation of pollen types based on growth habit assisting pre-screening of unknown pollen. 

### 3.2. Taxonomic Discrimination

Herb taxa were identified using PCA-SVM on the preprocessed Raman data. Four different herb pollen samples originating from four different families and genus were measured. Mean spectra of different herb pollen reveal different Raman bands (Figure 4a), which implies that these samples have different compositions of macromolecular content, e.g., the presence of 783 cm^−1^ (U,T,C ring breathing), and the 1441 cm^−1^ (CH_2_ scissor), 1660 cm^−1^ (amide I) and 2851 cm^−1^ (CH_2_ symmetric stretching) correlates mainly to lipids, whereas 1004 cm^−1^ (ring vibrations of phenylalanine) corresponds to protein features with minor contributions. Bağcıoğlu et al. also observed Raman band at 1173 cm^−1^, 1209 cm^−1^,1590 cm^−1^, 1610 cm^−1^, and 1637 cm^−1^ correlating it to mainly sporopollenins, a bio-chemical polymer which is found mainly in the outer wall of pollen grains, where he also connected protein to 1660 cm^-1^, 1455 cm^−1^, 1005 cm^−1^ and lipid to 1750 cm^−1^, 1444 cm^−1^, 1304 cm^−1^, 1065 cm^−1^ [2]. These spectral features also exist in the presented Raman spectra of this work. As band analysis is a great way to estimate macromolecular changes, multivariate analysis can provide a deeper insight into the underlying structure of the dataset. The first nine components of PCA correspond to total cumulative variance of 98.15%. As it is hard to visualize the high dimensional data, i.e., nine scores obtained from PCA, t-distributed stochastic neighbor embedding (t-SNE) was applied to the scores of the training dataset resulting in good separation between the samples (Figure 4b). t-SNE reduces the dataset dimension aiding the visualization for the high dimensional data [48], using the Barnes–Hut implementation [49]. The median values of t-SNE scores, represented by the big circles, provide better visualization of the separation in between different herb taxa (Figure 4b). Although the herb genera are separated using the first 2 PCs (Figure S1b), scores of other pollen samples from different growth habits overlap. The first five loadings of the PCA are presented in Figure S1c. Ten-fold cross-validation is performed resulting in an average misclassification of 3.2% for the PCA-SVM model of the herb sample. The prediction of genera in the test set resulted in an overall accuracy of 95.1% and is illustrated in a confusion matrix (Figure 4c). The overall sensitivities of the herb pollen samples are above 93%, presented in a graphical form (Figure 4d) for better visualization.

**Figure 5:**
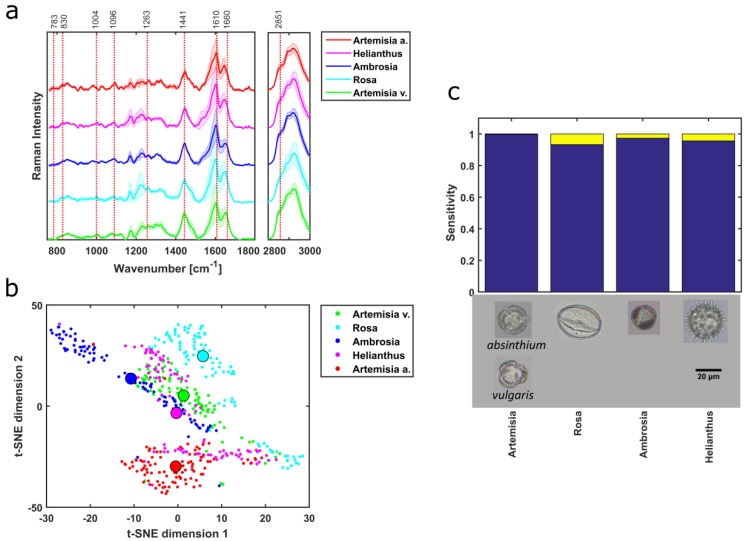
Background corrected mean spectra of six shrub pollen samples show distinct spectral features, including a strong influence at 1610 cm^−1^ related to sporopollenins (**a**). t-SNE plot of the corresponding PCA scores for training SVM model provides good separation in between the shrub genus as well as two species of *Arthmisia* (**b**). A sensitivity of over 95% for all the shrubs pollen samples was achieved after the PCA-SVM prediction (**c**).

Six different shrub pollen samples originating from three different families and five different genera were investigated. Two shrub samples share the same genus but different species, *Artemisia vulgaris* and *Artemisia Absinthium*. As before, different shrub samples are distinguished from their Raman spectral features (Figure 5a). The first nine PCA components correspond to total cumulative variance of 98.11 % and were later used for SVM modeling. The application of t-SNE to the scores of the shrub samples provides good separation in spite of some overlaps (Figure 5b). The t-SNE score median value of two genera, *Helianthus* and *Rosoideae*, are close to each other but separable (Figure 5b). Interestingly, the two species *Artemisia vulgaris* and *Artemisia absinthium* are nicely separated, confirming that Raman spectral information is capable to identify pollen on the species level. The different PCA loadings of the shrub (Figure S2c) compared to herbs (Figure S1c) affirm that both of the types constitute different spectral features and basis for each sample separation. Hence, they can be identified easily based on Raman spectral features. The ten-fold leave one out cross-validation on the trained PCA-SVM model has an average misclassification of 2.8%. The PCA-SVM predictions of shrub samples resulted in an accuracy of 96.9%. Four out of five shrub samples can be identified with sensitivities above 93% (Figure 5c). In the six shrub samples investigated, five geneses were present, leading to five predicted classes (Figure 5c and Figure S2d). Due to the large size, the confusion matrix is presented in (Figure S2d).

**Figure 6:**
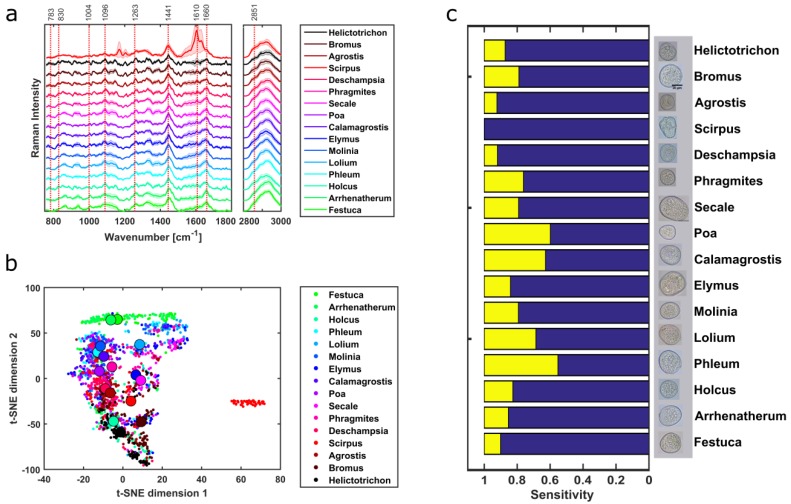
Background-corrected mean spectra of 16 grass pollen samples have similar spectral features as they share the same family except for *Scirpus* (**a**). t-SNE plot of the PCA scores for SVM training provides separation in between the grass genus, but many of their median as indicated by the big circles are placed close to each other (**b**). A sensitivity of around 80% for all the grass pollen samples was achieved after the PCA-SVM model prediction except for *Phleum*, *Calamagrostis,* and *Poa* (**c**).

Sixteen different grass pollen genera from two families were measured and identified using PCA-SVM on Raman spectra. Although the background-corrected spectra are quite similar, some distinct spectral features of the grass samples exist, e.g., between 1263 and 1441 cm^−1^ (Figure 6a) related to the different macromolecular composition mainly sporopollenins and lipids similar to [2]. *Scirpus* belongs to a different family than the rest of the grass samples and shows very different Raman spectra than the others. The first nine components of PCA correspond to total cumulative variance of 95.68%. The t-SNE plot of the grass pollen scores for the training dataset provides separation (Figure 6b), although some overlaps are present. Moreover, the median values of the t-SNE scores are also close to each other (Figure 6b) as 15 grass samples out of 16 are from the same family except *Scirpus,* which is quite well separated from the others. A spectral similarity of pollen having the same growth habit type has been previously reported [17,27]. The PCA loadings of the grass samples can be found in Figure S4c. The ten-fold leave one out cross-validation on the trained PCA-SVM model has an average misclassification of 22.13%. The predictions of the grass genus provided an accuracy of 78.78%. Most of the grass samples provided reasonable sensitivities around 80 % except for *Phleum*, *Calamagrostis,* and *Poa* (Figure 6c), whose influence is already evident in the accuracy. A closer look at the grass specimen morphologies depict clearly indicates the similarities among them and hence it posts a huge challenge for the image processing-based methods (Figure S5). Further details about the accuracy, sensitivity and positive predictive value are presented in the confusion matrix (Figure S4d).

**Figure 7:**
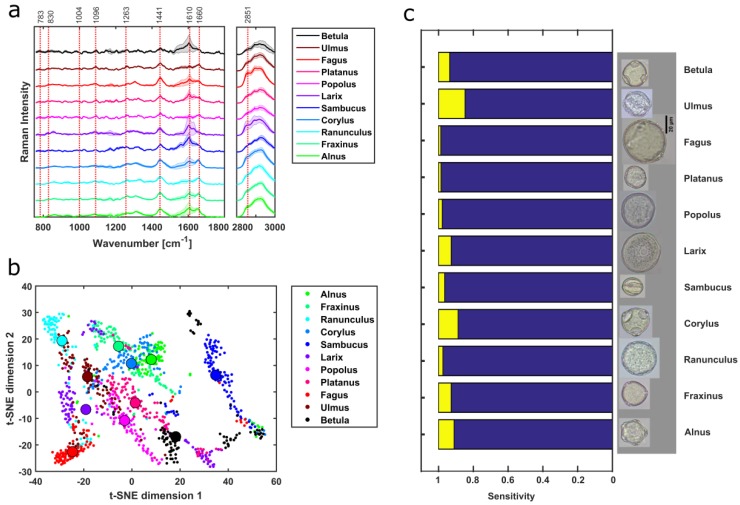
The background-corrected mean spectra of 11 tree pollen samples differ from each other at various Raman peak positions (**a**). t-SNE plot of the PCA scores for SVM training provides good separation between the tree genus (**b**). A sensitivity of over 90% for all the tree pollen samples was achieved after the PCA-SVM prediction except for *Ulmus* and *Corylus* (**c**).

Eleven different tree genera originating from nine families were identified using Raman spectra and PCA-SVM model. The background-corrected spectra show the potential of identifying the tree samples based on distinctive spectral signatures (Figure 7a) already mentioned above. The first nine components of PCA correspond to total cumulative variance of 97.19 %. The tree pollen t-SNE scores from training dataset clusters in different regions (Figure 7b). The generated PCA-SVM model from the training dataset has an average misclassification of 4.91% after ten-fold leave one out cross-validation. PCA-SVM prediction of tree genus has an accuracy of 94.05% with more than 90% of sensitivity except for *Ulmus* and *Corylus* (Figure 7c). Further analysis results are presented in detail using the confusion matrix (Figure S3d).

## 4. Discussion

The presented automated high throughput Raman spectroscopy (HTS-RS) platform allows to increase the throughput for characterization of pollen, with good genus estimation, fulfilling major demands of automated pollen detection [7,8]. Previous publications for pollen studies classified 3 to 15 different genera [2,5,17] as well as species [21,23,25] using Raman spectroscopy. Guedes et al. measured 34 pollen species from 18 families acquiring three to six pollen spectra from each of the samples [6]. In contrast, the here presented dataset contains approx. 22000 individual pollen grains from 18 families, 36 genera, and 37 species. Integration of customized pollen detection algorithm, improved autofocus function, homogenous illumination scheme, and fixed frames numbers for measurement improved the performance of HTS-RS, reducing the human dependency. Subgrouping of pollen into sub-category based on growth habit, i.e., grass, herb, shrub, and tree, is not common but has been shown before [6,27], where the subgrouping was performed based on previous knowledge. In this work, the application of HCA to Raman spectra provides a preliminary estimation of the pollen growth habit types. The grass sample identifications are more distinctive than the others (Figure 3a) because they originate from the same family as well as have similar spectral features (Figure 6a). HCA was also previously applied to pollen FTIR spectra resulting in accuracy of 84% [4], comparable to our HCA model with 75.7%. Supervised machine learning method PCA-SVM identified pollen genus with good accuracies and sensitivities except for grass samples. The spectral similarity of pollen from the same family was previously reported [17,27] and could be the reason for less accurate PCA-SVM prediction. For example, the mean spectra of *Festuca* and *Arrhenatherum* from the grass subtype have similar spectral features (Figure 6a) and are positioned next to each other in the HCA cluster tree (Figure 3a) as their spectral distance is minimal and their median t-SNE score are very close to each other (Figure 6b). Here, although the grass pollen specimens challenge the image-processing-based methods due to their similar morphological features (Figure S5), they are well identified using their corresponding Raman spectra, hence addressing a great problem of automated pollen detection. In shrubs two of the samples *Helianthus* and *Rosa* also have similar parameters, e.g., similar spectral features (Figure 5a), median t-SNE scores placed close to each other (Figure 5b) and similar nature in HCA cluster tree (Figure 3a). Additionally, the median value of *Artemisia vulgaris* and *Artemisia absinthium* t-SNE scores are well separated (Figure 5b). Different pollen taxonomies have been investigated using band analysis [5,20,21,26,27], hit quality and Euclidian distance [6], PCA [2,5,17,24], HCA [5,17,23,24] and artificial neural network (ANN) [24]. Seifert et al. showed that supervised methods are more effective than unsupervised ones, which is similar to our results [24]. ANN rendered an accuracy of 97% for 14 samples whereas PCA-ANN correctly identified 91%–92% of the samples [24]. Some other image-based pollen taxonomical analysis rendered 77% to 100% accuracy [9,11] but have identification challenges for pollen with similar size and texture, which are considered as major parameters for pollen detection [10,12], and can be easily addressed using Raman spectroscopy. Moreover, Raman analysis sheds light on complete macromolecular information. Application of HCA to FTIR pollen data provided 84% accuracy for 12 samples [4], but limits the measurement to the air environment only, but is acknowledged and demonstrated by HTS-RS method provide similar accuracy. Our results suggest that Raman spectral information can distinguish pollen with similar morphological features easily with high sensitivity, accuracy, and hence provide robustness. Although the bright-field images are used for cell detection and localization purpose, later combination of image-based analysis similar to previous works [7,9,10,11,12] with Raman spectroscopy would provide extensive morphological and chemical characterization particularly in more complex samples such as soil or air samples where a great concentration of other particles co-exist.

## 5. Conclusions

The HTS-RS has extended potential applications of conventional Raman spectroscopy systems by incorporating automation. With the HTS-RS, high-throughput and reproducibility are possible as it performs measurements fast in comparison to the conventional Raman measurement systems, without human interaction and label-free. Our results indicate that the HTS-RS offers large volume sampling, consistency, and good taxonomical resolution for pollen, and, most importantly, reliable discrimination of pollen specimens (e.g., the grass pollen) sharing similar morphological features, fulfilling the major needs of automated palynology and introducing it as an alternative tool.

## Figures and Tables

**Table 1:** List of investigated pollen samples specimens along with their family, genus, and type based on their growth habit.

Growth Habit	Family	Genus	Growth Habit	Family	Genus
Grass	Poaceae	Festuca	Shrub	Asteraceae	Artemisia a.
	Poaceae	Arrhenatherum		Rosaceae	Rosa
	Poaceae	Holcus		Asteraceae	Ambrosia v.
	Poaceae	Phleum		Asteraceae	Helianthus
	Poaceae	Lolium		Asteraceae	Artemisia
	Poaceae	Molinia			
	Poaceae	Elymus	Tree	Betulaceae	Alnus
	Poaceae	Calamagrostis		Oleaceae	Fraxinus
	Poaceae	Poa		Ranunculaceae	Ranunculus
	Poaceae	Secale		Betulaceae	Corylus
	Poaceae	Phragmites		Adoxaceae	Sambucus
	Poaceae	Deschampsia		Pinaceae	Larix
	Cyperaceae	Scirpus		Salicaceae	Populus
	Poaceae	Agrostis		Platanaceae	Platanus
	Poaceae	Bromus		Fagaceae	Fagus
	Poaceae	Helictotrichon		Ulmaceae	Ulmus
Herb	Primulaceae	Cyclamen		Betulaceae	Betula
	Polygonaceae	Rumex			
	Amaranthaceae	Amaranthus			
	Papaveraceae	Papaver			
	Plantaginaceae	Plantago			

## Supplementary Materials

The following are available online at https://www.mdpi.com/1424-8220/19/20/4428/s1, Figure S1: Herb pollen spectra before background correction and the results of PCA, Figure S2: Shrub pollen spectra before background correction, PCA and PCA-SVM results, Figure S3: Tree pollen spectra before background correction, PCA and PCA-SVM results, Figure S4: Grass pollen spectra before background correction, PCA and PCA-SVM results, Figure S5: Bright field images of Grass pollen samples.
