# Supplementary material for: Application of High-Throughput Screening Raman Spectroscopy (HTS-RS) for Label-Free Identification and Molecular Characterization of Pollen
Source: Sensors (Basel). 2019 Oct 12;19(20):4428. doi: 10.3390/s19204428 (PMC6832956; doi:10.3390/s19204428)
Supplement: Supplementary file 1 [file sensors-19-04428-s001.pdf]

sensors

MDPI

Supplementary Information

Application of High-Throughput Screening Raman Spectroscopy (HTS-RS) for Label-Free Identification and Molecular Characterization of Pollen

Abdullah S. Mondol [1,2], Milind D. Patel [1], Jan Rüger [1], Clara Stiebing [1], Andreas Kleiber [1], Thomas Henkel [1], Jürgen Popp [1,2] and Iwan W. Schie [1,3]*

[1] Leibniz Institute of Photonic Technology, Albert Einstein Str. 9, 07745 Jena, Germany; abdullah.mondol@leibniz-ipht.de (A.S.M.); patelmilind2993@gmail.com (M.D.P.); jan.rueger@leibniz-ipht.de (J.R.); clara.stiebing@leibniz-ipht.de (C.S.); andreas.kleiber@leibniz-ipht.de (A.K.); thomas.henkel@leibniz-ipht.de (T.H.); juergen.popp@ipht-jena.de (J.P.)

[2] Institute of Physical Chemistry and Abbe Center of Photonics, Friedrich-Schiller University Jena, Helmholtzweg 4, 07743 Jena, Germany

[3] Department of Medical Engineering and Biotechnology, University of Applied Sciences – Jena, Carl-Zeiss Promenade 2, 07745 Jena, Germany

* Correspondence: iwan.schie@leibniz-ipht.de; Tel.: +49-3641-206-313

Received: 29 August 2019; Accepted: 10 October 2019; Published: date

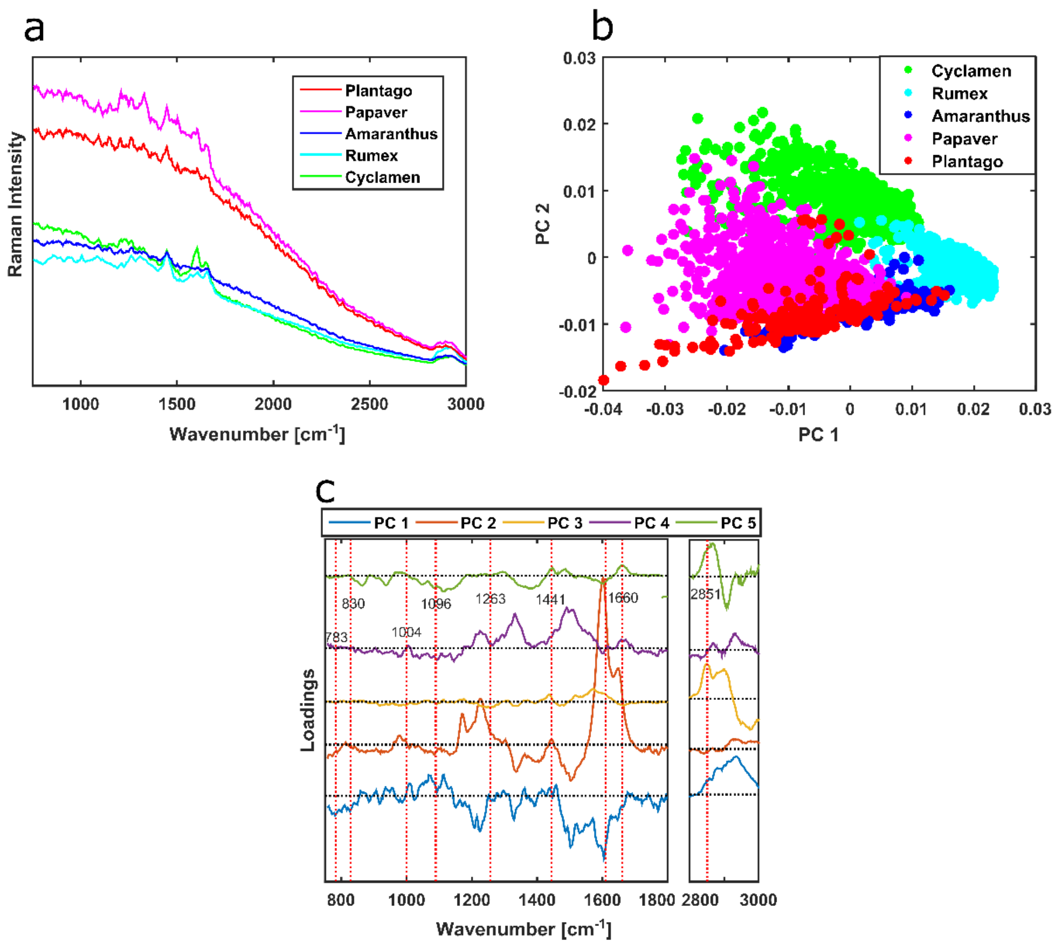

Figure S1. Mean Raman spectra of herb samples before background correction show high degree of fluorescence (a). The PCA scores of herb samples provide good separation considering only the first two PCs (b). The corresponding loadings of PCA are also presented (c).

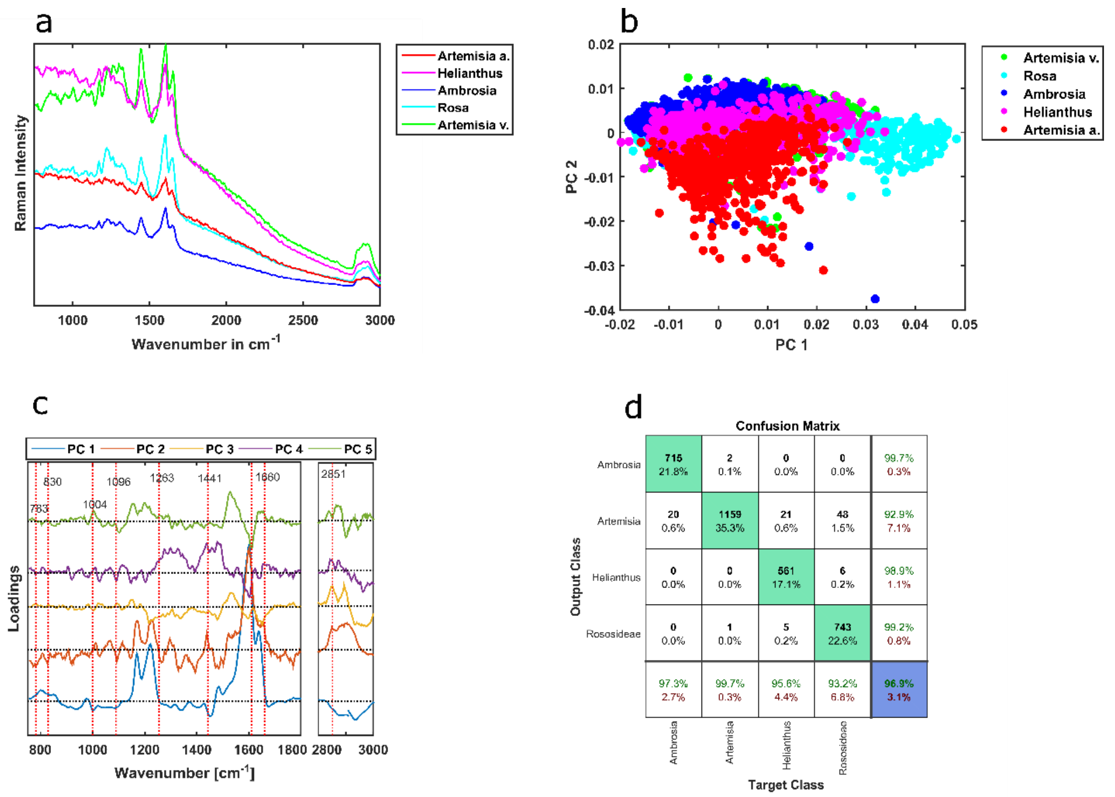

Figure S2. Non-background corrected mean Raman spectra of shrub samples constitute high degrees of fluorescence where two of them from same genus, *Artemisia* (a). The first two PCs hardly provide any separation of the sample, only showing overlaps (b) and hence, a t-SNE is required. The corresponding loadings of PCA are also presented (c).The summary of PCA-SVM prediction shows high accuracy and sensitivity as seen in the bottom row of the confusion matrix (d).

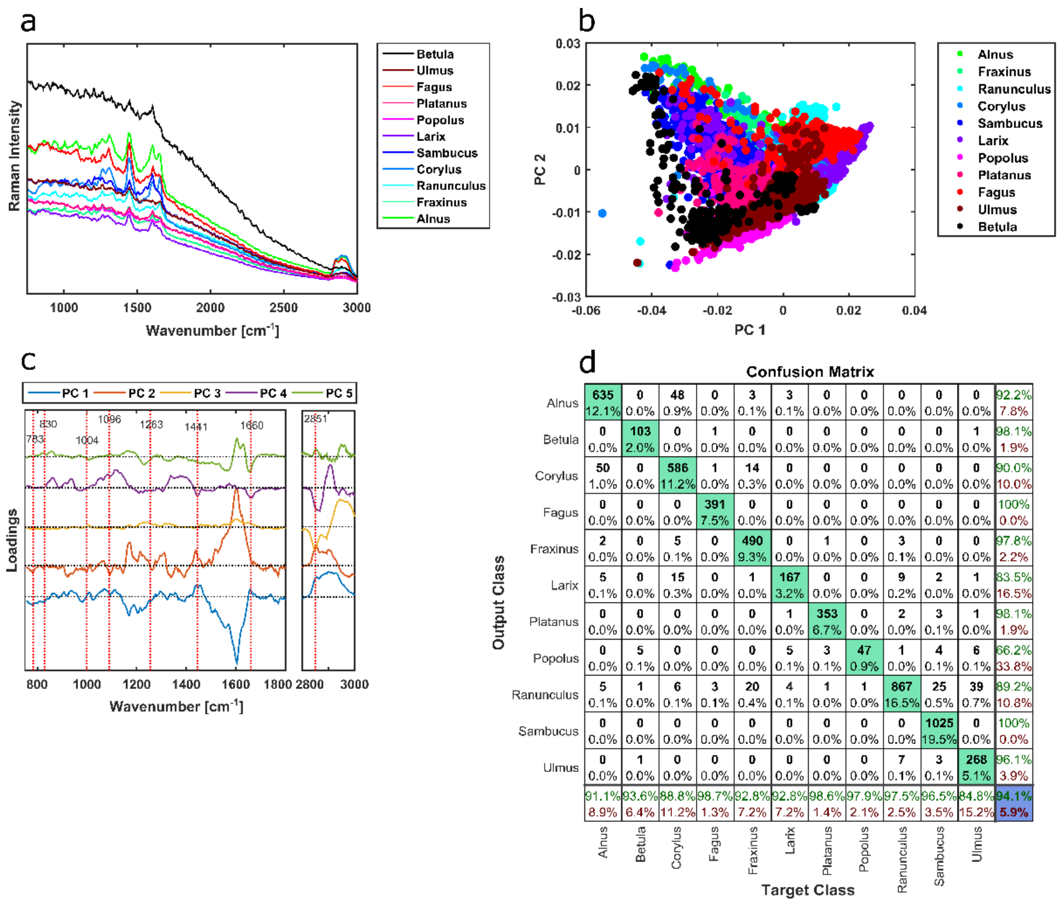

Figure S2. Unprocessed mean Raman spectra of eleven tree samples have high degree of fluorescence in it (a). The PCA scores of tree samples overlap with each other considering the first two PCs (b). The corresponding loadings of PCA are also presented in (c) correlating macromolecular changes to the PCs. The summary of PCA-SVM prediction shows high accuracy and sensitivity except for *Ulmus* and *Corylus* as indicated in the bottom row of the confusion matrix (d).

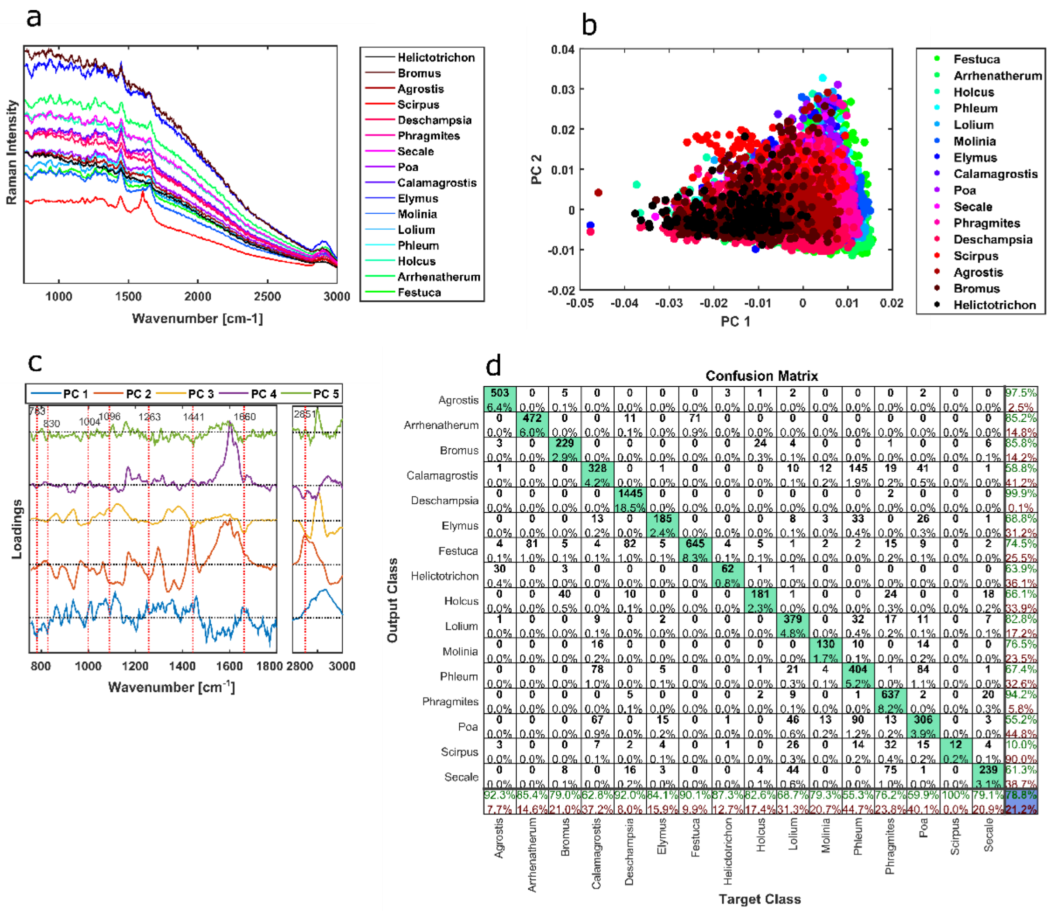

Figure S3. Mean spectra of 16 grass samples without background correction have high degree of fluorescence (a). The PCA scores of grass samples demonstrate high degree of overlap with each other while considering the first two PCs (b). The corresponding loadings of PCA are also presented (c).The confusion matrix of PCA-SVM prediction are summarized and it shows a moderate accuracy as well as sensitivity (d).

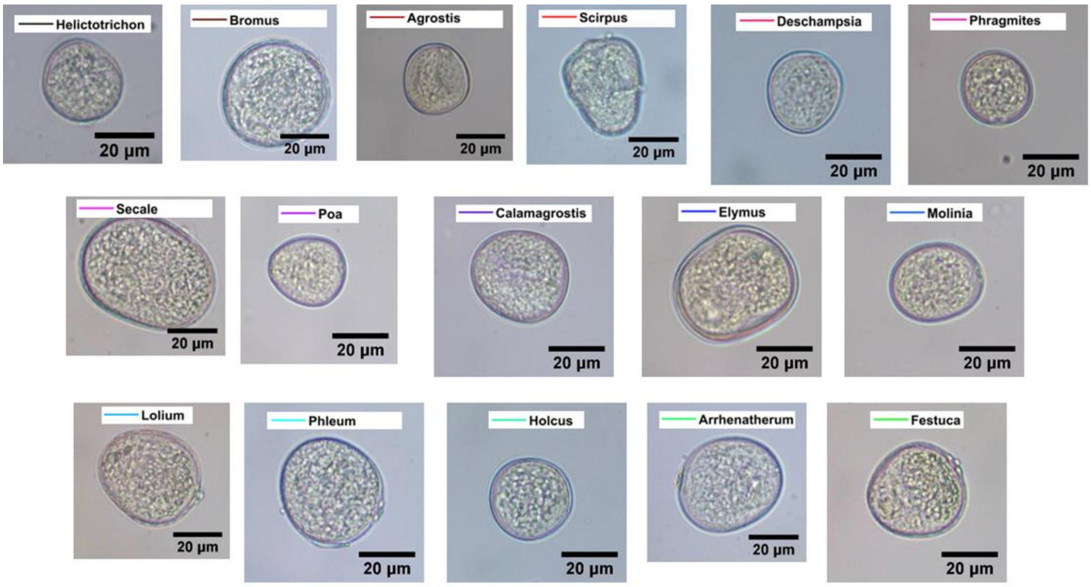

Figure S5. The bright field images of the investigated grass pollen samples show very similar morphological features considering the dimension and texture. By using Raman spectra they are easily identified. The colored line next to legends correlates the Raman spectra of figure 6a.
